# Epidemiology of respiratory syncytial virus in hospitalized children before, during, and after the COVID-19 lockdown restriction measures in Greece

**DOI:** 10.1017/S0950268824000724

**Published:** 2024-05-13

**Authors:** Maria M. Berikopoulou, Nick Dessypris, Elena Kalogera, Evangelia Petridou, Vasiliki Benetou, Levantia D. Zahariadou, Tania Siahanidou, Athanasios Michos

**Affiliations:** 1 School of Medicine, National and Kapodistrian University of Athens, Athens, Greece; 2 Department of Hygiene, Epidemiology and Medical Statistics, School of Medicine, National and Kapodistrian University of Athens, Athens, Greece; 3 Department of Microbiology, “Aghia Sophia” Children’s Hospital, Athens, Greece; 4 Department of Neonatology, First Department of Pediatrics, University of Athens, Athens, Greece; 5 Division of Infectious Diseases, First Department of Pediatrics, University of Athens, Athens, Greece

**Keywords:** Respiratory syncytial virus, RSV, COVID-19, Greece, children, seasonality, epidemiology, respiratory infection

## Abstract

The COVID-19 pandemic modified the epidemiology and the transmission of respiratory syncytial virus (RSV). We collected data on RSV positivity and incidence from children hospitalized in the largest tertiary paediatric hospital in Greece before (2018–2020, period A), during (2020–2021, period B), and after (2021–2023, period C) the COVID-19 lockdown. A total of 9,508 children were tested for RSV. RSV positivity (%) was 17.6% (552/3,134) for period A, 2.1% (13/629) for period B, and 13.4% (772/5,745) for period C (p < 0.001). The mean age (±SD) of RSV-positive children among the three periods was A: 5.9(±9.3), B: 13.6 (±25.3), and C: 16.7 (±28.6) months (p < 0.001). The peak of RSV epidemiology was shifted from January–March (period A) to October–December (period C). RSV in-hospital incidence per 1,000 hospitalizations in paediatric departments was A:16.7, B:1.0, and C:28.1 (p < 0.001), and the incidence in the intensive care unit was A: 17.3, B: 0.6, and C: 26.6 (p < 0.001). A decrease in RSV incidence was observed during the COVID-19 lockdown period, whereas a significant increase was observed after the lockdown. A change in epidemiological patterns was identified after the end of the lockdown, with an earlier seasonal peak and an age shift of increased RSV incidence in older children.

## Introduction

Respiratory syncytial virus (RSV) is an RNA virus belonging to the Paramyxoviridae family [1]. Although it can cause respiratory infections in all age groups, it is a major cause of bronchitis, bronchiolitis, and pneumonia in children, especially under 5 years of age [2, 3]. RSV is transmitted through human-to-human contact via respiratory droplets and can also be spread through dried respiratory secretions, with an incubation period ranging from 2 to 8 days [4].

In 2019, RSV was associated with 33 million cases of respiratory infections worldwide, leading to the hospitalization of 3.6 million children aged 0 to 60 months, with nearly 27,000 in-hospital fatalities [5]. The burden of RSV is exacerbated by factors such as prematurity, younger age, and low socioeconomic status [5–7].

Severe acute respiratory syndrome coronavirus 2 (SARS-CoV-2) caused a global pandemic with significant morbidity and mortality [8–10]. To contain the transmission of SARS-CoV-2, non-pharmaceutical interventions such as social distancing, mask-wearing, and lockdowns were implemented [11]. These interventions not only reduced the spread of SARS-CoV-2 but also had a notable impact on the transmission of other respiratory viruses like RSV [12]. Due to the COVID-19 restrictions, off-season RSV epidemics have been observed [13, 14].

In Greece, the first COVID-19 patient was diagnosed on 26 February 2020. The Greek government suspended educational institutions on 11 March 2020. Stricter measures were implemented, leading to a general lockdown on 23 March 2020. After experiencing a second wave of the pandemic, educational institutions were eventually reopened in April 2021 [15].

Before the COVID-19 pandemic and the suspension of educational institutions, RSV transmission in Greece typically followed a seasonal pattern [16]. Currently, in Greece, the only monoclonal antibody available for high-risk infants to prevent RSV infection is palivizumab. Given the licensure of one-dose RSV monoclonal antibodies and RSV vaccines for pregnant women and adults [17], surveillance of data regarding RSV epidemiology could provide valuable guidance for public health decisions.

The objective of this study was to examine the potential change in RSV epidemiology in the period before, during, and after the COVID-19 lockdown restriction measures in a large hospitalized paediatric population in Greece.

## Materials and methods

A retrospective observational study was carried out at ‘Aghia Sophia’ Children’s Hospital in Athens, from 1 January 2018 to 1 May 2023. The research ethical clearance approval letter was obtained from the Research Ethics Committee at the ‘Aghia Sophia’ Hospital, in November 2023 with the protocol number 20818/21.09.2023. ‘Aghia Sophia’ is the largest paediatric hospital in Greece (750 beds), serving approximately 40% of the paediatric population in the Athens metropolitan area.

The study population consisted of all children from birth until 16 years of age who had been hospitalized during the study period. Children who were hospitalized with lower respiratory symptoms had been tested for RSV via a rapid antigen immune chromatography test in nasopharyngeal wash specimens (RSV Respi-Strip, Coris-Bioconcept). Data were retrieved from the microbiology laboratory archive. Children over 16 years, children with inadequate samples for testing, and children with duplicate tests within a month were excluded from the study. Additional data about the overall number of hospitalizations during the total study period from paediatric clinics and intensive care units (ICUs) were retrieved from the hospital’s statistical department.

The overall study period was divided into three subperiods based on the COVID-19 lockdown restriction measures: before (January 2018–February 2020, period A), during (March 2020–June 2021, period B), and after (July 2021–May 2023, period C) the COVID-19 lockdown.

For each period, RSV positivity rate (%) was calculated by dividing the total number of RSV-positive samples by the total number of samples examined in the laboratory during the respective time period. RSV in-hospital incidence per 1,000 hospitalizations was also calculated for each period by dividing the total number of RSV-positive samples by the total number of hospitalizations during the respective time period.

Subsequently, data from each period were divided into four subperiods: January–March (I), April–June (II), July–September (III), and October–December (IV). The corresponding positivity rate and incidence of RSV for each period were calculated and compared with each other. The data were also analysed for each age group separately to examine the possible effect of age on the observed differences between the subperiods and the comparisons of the positivity rates.

For statistical analysis, initially, Pearson’s chi-squared test or Fisher’s exact test was used to compare data between the study subperiods. An analysis of variance (ANOVA) test was conducted to compare the mean age between the three periods. Logistic regression was also performed to estimate the possible effect of children’s age and each subperiod on the RSV positivity rate.

Statistical significance was set at 0.05. Statistical analysis was conducted using SAS (v 9.4) statistical analysis software.

## Results

During the study period, a total of 10,647 children were tested for RSV. Among them, 1,139 children were excluded due to inadequate samples for testing or duplicate tests within a month. Finally, 9,508 children, with a mean (±SD) age of 21.7 (±33.6) months, were included in the analysis. Among them, 826 (8.7%) were newborns (<1 month), 4,851 (51.0%) were infants (1 month to 1 year), 2,058 (21.6%) were toddlers (>1 year to 3 years), 1,023 (10.8%) were of preschool age (>3 years to 6 years), 490 (5.2%) were of school age (>6 years to 11 years), and 260 (2.7%) were adolescents (>11 years to 16 years). The age distribution between the three periods was as follows: A: 3,134 children with a mean (±SD) age of 10.6 (±20.3) months, B: 629 children with a mean (±SD) age of 13.8 (±24.4) months, and C: 5,745 children with a mean (±SD) age of 28.6 (±38.3) months (p < 0.001). The increase in age across the three periods was further confirmed by logistic regression analysis, which showed that each additional year of age increased the likelihood of being in a later period by more than 50% (OR = 1.69, (95% CI = 1.47–1.95), p-value = 0.0001).

RSV positivity (%) during the total study period was 14.06% (1,337/9,508), and 741 (55%) were male. RSV positivity (%) during the three study periods was as follows: A: 17.6% (552/3,134), B: 2.1% (13/629), and C: 13.4% (772/5,745) (p < 0.001). The mean (±SD) age of RSV-positive children among the three study periods was A: 5.90 (±9.30), B: 13.55(±25.26), and C: 16.71 (±28.58) months (p < 0.001).

**Figure 1.**
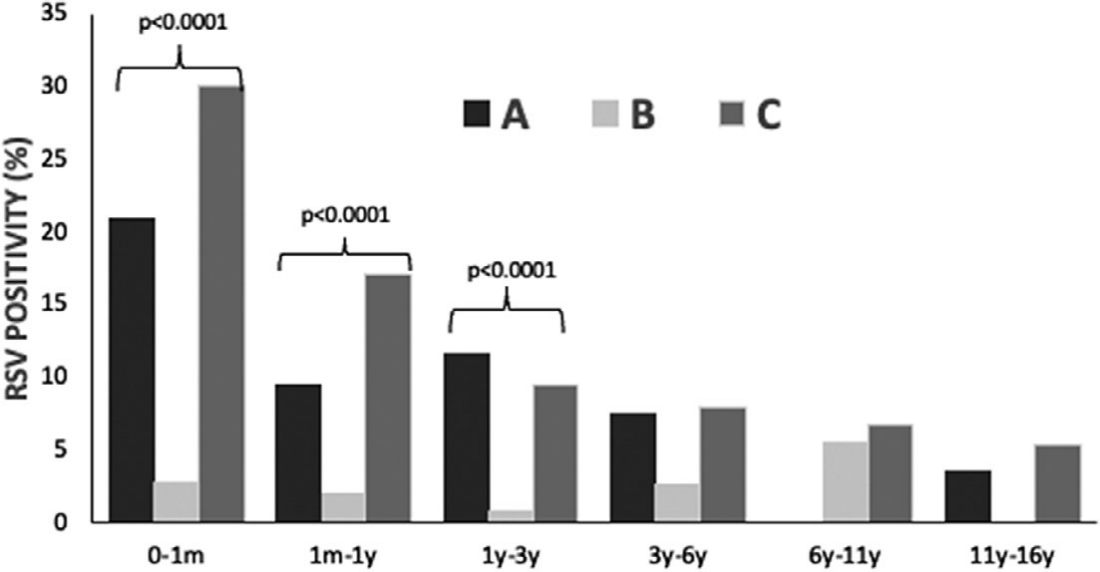
RSV infection positivity rate (%) in hospitalized children (n = 9508) before (January 2018 – February 2020, period A), during (March 2020 – June 2021, period B) and after (July 2021 – May 2023, period C) COVID-19 lockdown restrictions measures.

Specific RSV positivity (%) per age group is presented in Table 1 and Figure 1. Comparing the three study periods for each age group, statistically significant differences were detected for ages 0–1 month: A: 21.0%, B: 2.8%, and C: 30.1% (p < 0.001); 1 month–1 year: A: 9.5%, B: 2.1%, and C: 17.1% (p < 0.001); and 1 year–3 years: A: 11.7%, B: 0.9%, and C: 9.5% (p < 0.001). No statistically significant differences regarding RSV positivity were detected for age groups >3 years (3–6 years, 6–11 years, and 11–16 years).

**Table 1.** RSV positivity (%) before (January 2018–February 2020, period A), during (March 2020–June 2021, period B), and after (July 2021–May 2023, period C) COVID-19 lockdown restrictions measures per subperiods within different age groups in hospitalized children

		RSV positivity (%)			*P*-value
Age groups	Total number of positive children	Period A (*n* = 3,134)	Period B (*n* = 629)	Period C (*n* = 5,745)	
0–1 m	826	21 (81/385)	2.8 (2/72)	30.1 (111/369)	<0.001
1 m–1y	4,851	9.5 (402/2,058)	2.1 (8/379)	17.1 (414/2,414)	<0.001
1y–3y	2,058	11.7 (60/511)	0.9 (1/117)	9.5 (136/1,430)	0.001
3y–6y	1,023	9.7 (8/105)	2.7 (1/37)	7.9 (70/881)	0.500
6y–11y	490	0 (0/47)	5.6 (1/18)	6.8 (29/425)	0.180
11y–16y	260	3.6 (1/28)	0 (0/6)	5.3 (12/226)	0.790
0–16y (*n* = 9,508)	Subperiods				
	October–December	4.9	0	23.7	<0.001
	January–March	25.7	0	12.2	<0.001
	April–June	7.8	1.4	3.4	0.001
	July–September	2.1	4.7	2.3	0.860
	Total	17.6 (552/3,134)	2.1 (13/629)	13.4 (772/5,745)	<0.001

m, months; y, years.

RSV positivity (%) for the three study periods for each subperiod and for each age group is presented in Table 1. Before the COVID-19 lockdown (period A), the peak of RSV incidence was observed during January–March, with a positivity rate (%) of 25.7%, followed by April–June (7.8%). During the lockdown period (B), the positivity rate (%) during October–March was 0%, and the highest peak was detected during April–June (4.7%). Finally, after the COVID-19 lockdown (period C), the peak of RSV positivity was observed during October–December (23.7%).

Logistic analysis (Table 2) confirms the above findings. Specifically, during the pre-lockdown period, the primary peak of RSV positivity and associated excess of the virus, as compared to the lockdown period, occurred in the well-established time frame of January to March (OR = 16.31, 95% CI = 9.32–28.56, p-value = 0.0001), followed by April–June and September–December (OR = 3.27, 95% CI = 1.97–7.23, p-value = 0.0001; OR = 2.34, 95% CI = 1.22–4.50, p-value = 0.01, respectively). However, in the post-lockdown period, the highest peak of RSV positivity occurred during October–December (OR = 17.82, 95% CI = 10.17–31.21, p-value = 0.0001), followed by January–March (OR = 8.51, 95% CI = 4.82–15.02, p-value = 0.0001).

**Table 2.** Logistic regression analysis of RSV positivity

Variable	Category	OR	95% CI		*P*-value
Period/subperiod					
	Pre–lockdown (April–June)	3.77	1.97	7.23	<0.0001
	Pre–lockdown (July–December)	0.92	0.26	3.27	0.90
	Pre–lockdown (October–December)	2.34	1.22	4.50	0.01
	Pre–lockdown (January–March)	16.31	9.32	28.56	<0.0001
	Lockdown	Baseline			
	Post–lockdown (April–June)	2.27	1.21	4.28	0.01
	Post–lockdown (July–September)	1.24	0.58	2.66	0.59
	Post–lockdown (October–December)	17.82	10.17	31.21	<0.0001
	Post–lockdown (January–March)	8.51	4.82	15.02	<0.0001
Age	One category more	0.64	0.59	0.68	<0.0001

**Figure 2.**
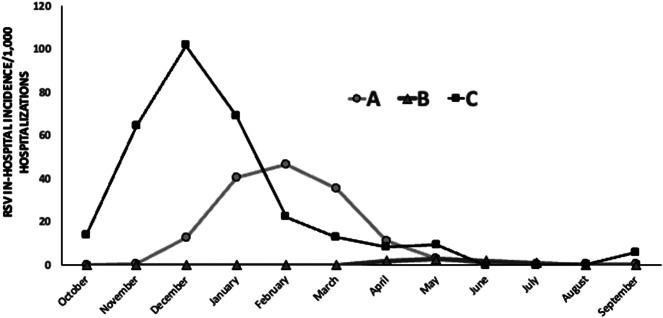
RSV in-hospital incidence (per 1000) in hospitalized children (n = 9508) before (January 2018 – February 2020, period A), during (March 2020 – June 2021, period B) and after (July 2021 – May 2023, period C) COVID-19 lockdown restrictions measures.

RSV in-hospital incidence per 1,000 hospitalizations for each month throughout the study period is presented in Figure 2 and Supplementary Figure S1 (available on the Cambridge Core website). RSV in-hospital incidence for the total study period was 18.1/1,000 hospitalizations. A statistically significant difference in in-hospital RSV incidence per 1,000 hospitalizations was detected between the three study periods: A: 16.7 (552/33,131), B: 1.0 (13/13,079), and C: 28.1 (772/27,509) (p < 0.001).

The peak of RSV in-hospital incidence before the COVID-19 lockdown (period A) occurred during January–March at a rate of 41.3/1,000 hospitalizations, followed by April–June at 4.9/1,000 hospitalizations. During the COVID-19 lockdown (period B), RSV in-hospital incidence during October–March was 0% (p < 0.001), and a low peak was detected during April–June (2.2/1,000 hospitalizations). After the COVID-19 lockdown, the peak of RSV in-hospital incidence was observed during October–December (61.9/1,000 hospitalizations) (p < 0.001). RSV in-hospital incidence for the three periods is presented in Supplementary Table S1 (available on the Cambridge Core website).

**Figure 3.**
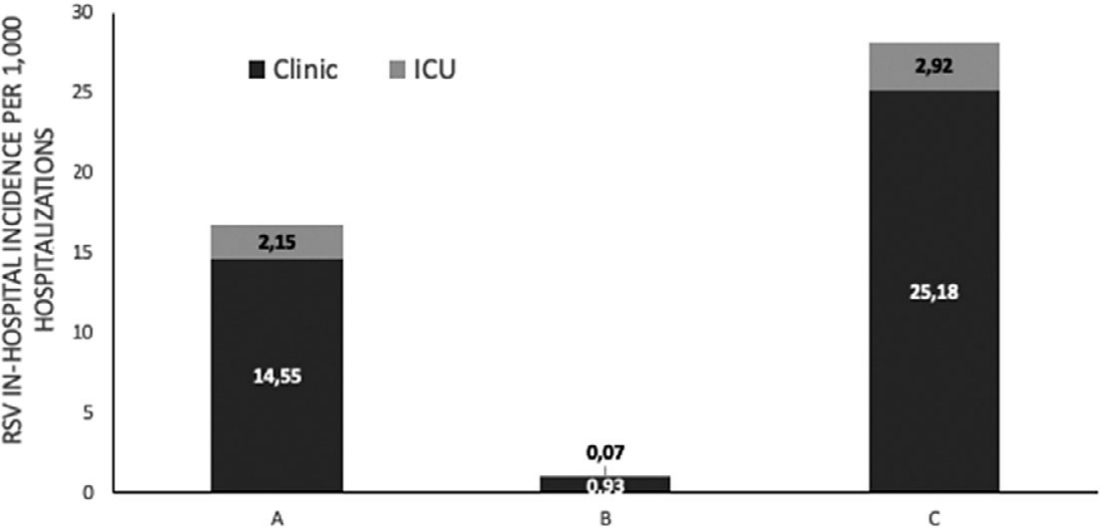
RSV in-hospitalized incidence per 1000 hospitalizations in general pediatric department and neonatal or pediatric intensive care unit ICU (%), before (January 2018 – February 2020, period A), during (March 2020 – June 2021, period B) and after (July 2021 – May 2023, period C) COVID-19 lockdown restrictions measures.

Among RSV-positive children, 151/1,337 (11.29%) required admission in the neonatal intensive care unit (NICU) or paediatric intensive care unit (PICU) and 56% were male (84/151). RSV infection incidence per 1,000 hospitalizations in the general paediatric department and NICU or PICU (%) is shown in Figure 3. Among them, 133 (88%) children were of 0–1 month of age, 13 (8.6%) were of 1 month–1 year of age, 4 (2.6%) were of 1–3 years of age, and 1 child (0.6%) was older than 3 years. The distribution of children who were admitted to the ICU due to RSV infection among the three study periods was A: 70/3,134 (2.23%) children, B: 1/629 (0.16%) children, and C: 80/5745 (1.4%) children (p < 0.001). Their mean (±SD) age for the three study periods was A: 2.0 (± 6.5) months, B: 0.2 (N/A) months, and C: 1.3 (±4.1) months (p = 0.7). Among ICU admissions, RSV positivity (%) among children admitted to the ICU during the three study periods was A: 18.0% (70/389), B: 1.6% (1/61), and C: 25.5% (80/314) (p < 0.001), whereas RSV incidence per 1,000 hospitalizations in the ICU was A: 17.3, B: 0.6, and C: 26.6 (p < 0.001).

## Discussion

In the present study, we described the epidemiology of RSV before, during, and after COVID-19 lockdown restriction measures in a large sample of children hospitalized in the largest paediatric hospital in Athens, Greece. RSV incidence was found to be decreased during the COVID-19 lockdown period, whereas it increased significantly after the lockdown period, and a shift was detected in RSV epidemiology involving mostly older children and an earlier peak than in usual periods.

Similar reports from Finland, Italy, the UK, and the USA indicate a significant decline in RSV incidence during the SARS-CoV-2 restriction measures, compared with previous years [18–22]. In Western Australia, a decline of up to 98% was also detected in RSV epidemiology during the winter of 2020 [23]. This reduction in RSV incidence could most probably be attributable to the total lockdown combined with non-pharmaceutical interventions, such as following hand hygiene practices and using face masks [9]. After the reduced circulation of RSV, an increased incidence and positivity were observed worldwide [21, 22, 24]. This could be attributable to the increased circulation of the virus after the end of restriction measures or to the absence of immunity to the virus, which could make children more vulnerable [14, 25].

Additionally, there was a shift in RSV seasonal patterns. A multi-centre analysis across 11 countries reported a consistent delay in RSV peak, ranging from 13 weeks in France to 88 weeks in Brazil, with an average delay of 39 weeks. These delayed seasons were characterized by high RSV activity outside the normal period: summer instead of winter in South Africa, the Netherlands, Israel, and the USA; and 13 weeks later in winter instead of late autumn in France [26, 27]. A very recent study at two large Austrian paediatric departments also noticed an earlier RSV peak; in the first centre in November and in the second one in October [28]. This shifted epidemiology could be due to a viral interference effect and the ongoing use of face masks and other non-pharmaceutical interventions. A similar effect was detected during the 2009 H1N1 influenza pandemic, where RSV peak and seasonality were delayed up to 2.5 months [24].

When we compared the mean age of RSV-positive children between the three periods of our study, an age shift to older children was observed, even though the majority of RSV-positive children were under 3 years of age. In most studies, RSV posed a higher risk and necessitated hospitalization in infants, while older children typically exhibited milder symptoms [6]. A study carried out across seven different hospitals in the USA reported that 87% of RSV-positive children were under 2 years of age [29]. Nevertheless, data from Iceland indicated that an age shift in RSV-positive children also occurred during the pandemic, and the median age of RSV-positive cases increased from 5.7 months to 16 months in 2020–2021 [30].

After the COVID-19 restriction measures, a significant increase in RSV-positive children requiring admission to the PICU was observed. A study conducted in British Columbia, Canada, from 1 September 2017 to 15 May 2023 also showed a considerable increase in children up to 6 months of age after COVID-19. The severity of RSV infection increased due to more children requiring supplemental oxygen after the restriction measures. However, the proportion of children who required mechanical ventilation and the number of deaths did not change [22]. Another study among four different Italian hospitals showed an increased admission rate to the ICU after the COVID-19 period from 18% to 29% (p = 0.013) [20]. However, in contrast to these findings, data from two Austrian hospitals indicated that, although there was an increase in the number of RSV-infected children, no significant change in the admission rate to the PICU or in the mortality rate was detected [28].

Results from this study are based on a large sample of children hospitalized in the largest tertiary paediatric hospital in Athens, Greece. However, as they are single-centre data, they cannot be generalized to the whole population of children. The calculated incidence represents the in-hospital incidence, which indicates the RSV hospital burden; however, it is an underestimation of the population burden. In addition, data regarding the duration of hospitalizations, the possible nosocomial transmission, and therapeutic interventions needed were not available from the electronic records in order to have additional criteria for the evaluation of clinical severity.

Data presented in the present study indicate a significant increase in the incidence of RSV infection after the SARS-CoV-2 restriction measures, with more ICU admissions and shifted epidemiological patterns in the study sample. Given the recent availability of RSV vaccines and one-dose RSV monoclonal antibodies, surveillance of data regarding RSV epidemiology is important and could provide valuable data that could guide the decisions of public policymakers and authorities. It would be intriguing to conduct further research to observe whether the seasonal epidemiology of RSV will return to its previous patterns in the upcoming years.

## Supporting information

Berikopoulou et al. supplementary material Berikopoulou et al. supplementary material

## Data Availability

The data presented in this study are available on request from the corresponding author. The data are not publicly available due to anonymity and confidentiality.

## Supplementary material

For supplementary material accompanying this paper visit http://doi.org/10.1017/S0950268824000724. click here to view supplementary material
