# Supplementary material for: Epidemiology of respiratory syncytial virus in hospitalized children before, during, and after the COVID-19 lockdown restriction measures in Greece
Source: Epidemiol Infect. 2024 May 13;152:e94. doi: 10.1017/S0950268824000724 (PMC11736444; doi:10.1017/S0950268824000724)

Epidemiology and Infection

Epidemiology of Respiratory Syncytial Virus in hospitalized children before, during and after the COVID-19 lockdown restriction measures in Greece

Maria M. Berikopoulou, Nick Dessypris, Eleni Kalogera, Evi Petridou, Vasiliki Benetou, Levantia D. Zahariadou, Tania Siahanidou, Athanasios Michos

***Supplementary Table 1:*** RSV in-hospital incidence per 1000 hospitalizations, before (January 2018 – February 2020, period A), during (March 2020 – June 2021, period B) and after (July 2021 – May 2023, period C) COVID-19 lockdown restrictions measures per subperiods in hospitalized children.

|  | | **In- hospital incidence per 1000 hospitalizations** | | | **P-value** |
| --- | --- | --- | --- | --- | --- |
| **Age** | **Subperiods** | **Period A** | **Period B** | **Period C** |  |
| **0-16 years**  **(n=73719)** | October-December | 4.4  (33/7508) | 0  (0/2586) | 61.9  (484/7822) | <0.001 |
|  | January-March | 41.3  (481/11650) | 0  (0/2387) | 33.6  (233/6930) | <0.001 |
|  | April-June | 4.9  (35/7087) | 2.2  (12/5400) | 6.9  (41/5939) | 0.001 |
|  | July-September | 0.4  (3/6886) | 0.4  (1/2706) | 2.1  (14/6818) | 0.007 |
|  | **Total** | 16.7  **(552/33131)** | 1.0  **(13/13079)** | 28.1  **(772/27509)** | <0.001 |

***Supplementary Figure 1:*** RSV in-hospital incidence per 1000 hospitalizations in hospitalized children (n=9508) from January 2018 to May 2023


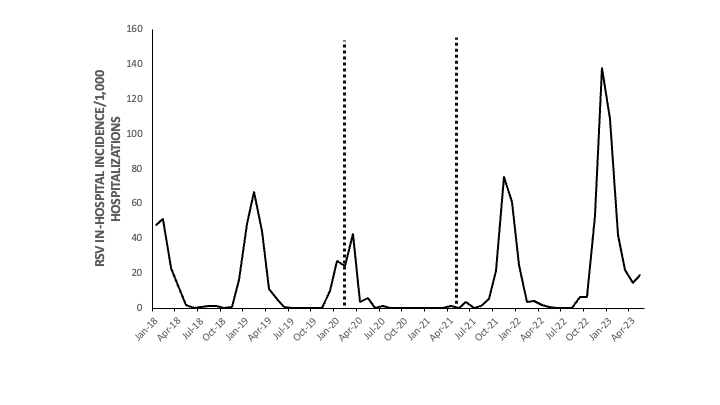

Supplement: Berikopoulou et al. supplementary material [file S0950268824000724sup001.docx]
